# Monitoring metallic sub-micrometric lithium structures in Li-ion batteries by in situ electron paramagnetic resonance correlated spectroscopy and imaging

**DOI:** 10.1038/s41467-021-21598-2

**Published:** 2021-03-03

**Authors:** Charles-Emmanuel Dutoit, Mingxue Tang, Didier Gourier, Jean-Marie Tarascon, Hervé Vezin, Elodie Salager

**Affiliations:** 1 CNRS, CEMHTI UPR3079, Université d’Orléans, Orléans, France; 2 Réseau sur le Stockage Electrochimique de l’Energie (RS2E), FR CNRS 3459, Amiens Cedex, France; 3 Chimie-ParisTech, PSL Université, CNRS, Institut de Recherche de Chimie-Paris (IRCP), Paris, France; 4 Collége de France, CNRS FRE3357, Paris, France; 5 Université Lille Nord de France, CNRS UMR8516, LASIRE, Villeneuve d’Ascq, France

**Keywords:** Batteries, Imaging techniques

## Abstract

Monitoring the formation of dendrites or filaments of lithium is of paramount importance for Li-based battery technologies, hence the intense activities in designing in situ techniques to visualize their growth. Herein we report the benefit of correlating in situ electron paramagnetic resonance (EPR) spectroscopy and EPR imaging to analyze the morphology and location of metallic lithium in a symmetric Li/LiPF_6_/Li electrochemical cell during polarization. We exploit the variations in shape, resonance field and amplitude of the EPR spectra to follow, operando, the nucleation of sub-micrometric Li particles (narrow and symmetrical signal) that conjointly occurs with the fragmentation of bulk Li on the opposite electrode (asymmetrical signal). Moreover, in situ EPR correlated spectroscopy and imaging (spectral-spatial EPR imaging) allows the identification (spectral) and localization (spatial) of the sub-micrometric Li particles created by plating (deposition) or stripping (altered bulk Li surface). We finally demonstrate the possibility to visualize, via in situ EPR imaging, dendrites formed through the separator in the whole cell. Such a technique could be of great help in mastering the Li-electrolyte interface issues that plague the development of solid-state batteries.

## Abstract (web summary)

Monitoring the nucleation of dendrites in Li-ion batteries during cell cycling is important for the development of new electrochemical materials. Here, the authors use the spectral-spatial mode in electron paramagnetic resonance imaging to visualize the spatial distribution of metallic sub-micrometric lithium structures.

## Introduction

Rechargeable lithium-ion batteries (LIBs) are recognized for the good balance between weight, volume, and electrochemical performance. Despite the wide range of applications for LIBs, prevention of non-uniform deposition of metallic lithium is still of paramount importance to avoid internal short-circuits and thermal runaway. Formation of lithium dendrites on the negative graphitic electrode is an issue in mature LIBs that prevents faster charge, essential for the development of transport electrification^1,2^, while lithium filament growth hinders the spread of all-solid-state batteries^3,4^.

Metallic lithium deposition in batteries is still relatively hard to predict, and the visualization, in real time, of its location, morphology and growth is the subject of intense development for many techniques^5^. A precious feature of electron paramagnetic resonance (EPR) is the combination of imaging and spectroscopy, unexplored to date for the characterization of lithium deposition in batteries. In 2015 in situ EPR was demonstrated as a tool of choice to detect Li deposits, either by imaging^6^ or spectroscopy^7^. In continuation of the latter an extensive study by operando EPR spectroscopy was performed for Li plating on graphite^8^. Niemöller et al. also studied, ex situ, the potential and limitations of ex situ EPR imaging (EPRI) on metallic Li^9^.

In this paper, we report the combination of in situ X-band EPR spectroscopy and imaging to observe directly, in situ, the morphology and the distribution of metallic lithium in a symmetric Li/LiPF_6_/Li electrochemical cell. We observe the roughening of the lithium surface upon stripping, the spatially inhomogeneous electrodeposition of the sub-micrometric lithium particles and dendrite growth causing a short-circuit.

## Results and discussion

**Fig. 1:**
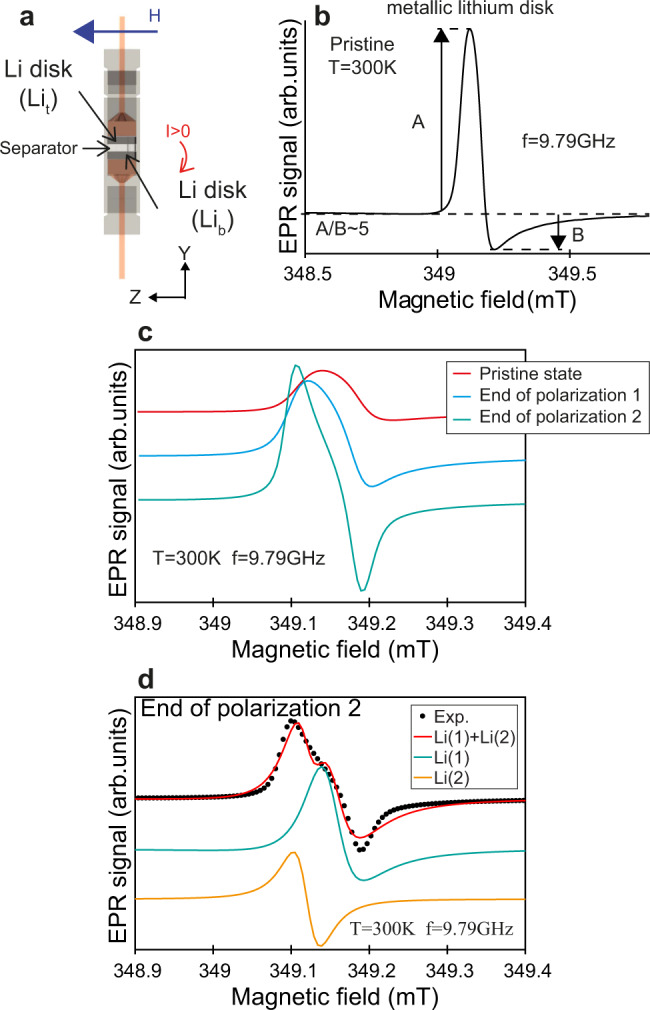
Operando EPR spectroscopy recorded at 9.79 GHz. **a** Schematic representation of the EPR electrochemical cell and its orientation in the external magnetic field **H** created by Charles-Emmanuel Dutoit using an open-source 3D computer graphics software (Blender 3D). **Y** and **Z** are the magnetic field gradient directions for imaging. **b** cw-EPR spectrum of a pristine metallic lithium disk (no cycling). A and B denote the amplitude of the positive and negative parts of the EPR line, respectively. **c** Selection of operando cw-EPR spectra of the Li/LiPF_6_/Li cell. A symmetrical and sharp EPR line appears in the low magnetic field flank of the bulk signal upon polarization. **d** Experimental (black dots) and simulated (red line) EPR spectra at the end of polarization 2. The dysonian component of the simulation Li(1) is assigned to the bulk Li signal of both electrodes and the lorentzian component Li(2) to the newly formed sub-micrometric Li particles.

Figure 1a shows a schematic representation of the Li/LiPF_6_/Li electrochemical cell placed in the EPR resonator, with the static magnetic field **H** oriented along the **Z** axis and the microwave field along the **Y** axis. The magnetic field gradients, used for imaging, are oriented along the **Y** and **Z** axes. This configuration is optimized to study lithium exchange between the two electrodes and especially dendritic growth. The EPR spectrum of the assembled electrochemical cell in the pristine state, before applying the current flow, is shown in Fig. 1c. It is identical to the spectrum of a metallic lithium disk (Fig. 1b), as expected. The resonance field and, therefore, the measured *g* factor (*g* = 2.0030, close to the free electron spin *g* = 2.0023) is characteristic of metallic lithium.

Previous EPR investigations showed that the lineshape, linewidth and integrated intensity were highly dependent on the morphology of metallic lithium^6,7,9^. The EPR spectrum lineshape of a metallic conductor is indeed controlled by the limited penetration depth *δ*_*mw*_ of the microwave field in the metal, known as the skin effect^10^:

1 $${\delta }_{mw}\propto \sqrt{\frac{\rho }{f}}$$

*ρ* being the metal resistivity and *f* the microwave frequency.

More precisely, the EPR spectrum lineshape depends on the metal thickness *d* compared to *δ*_*mw*_. If *d* > *δ*_*mw*_, only the spins located inside the skin depth are excited by the microwave field. During the spin coherence time *T*_2_, each electron travels on a distance of the order of ${\delta }_{eff}\approx {({D}_{e}{T}_{2})}^{\frac{1}{2}}$ where *D*_*e*_ is the diffusion coefficient of electrons^10,11^. As *δ*_*eff*_ ≈ 60 μm in metallic lithium^9^, the electrons penetrate several times in the skin region during *T*_2_ (~10^−9^ s), and thus experience each time a microwave pulse, resulting in an asymmetrical (dysonian) lineshape classically characterized by the asymmetry ratio A/B ≈ 5^10,11^. If *d* < *δ*_*mw*_, all the electronic spins experience the same microwave field, resulting in a symmetrical Lorentzian EPR line. Consequently, the presence of sub-micrometric metallic lithium such as dendritic and/or mossy structures can be revealed by the shape of the EPR spectrum. As expected for a Li electrode in the pristine state (Fig. 1b), a single dysonian lineshape with A/B ≈ 5 is observed, typical of thick metal. This is in good agreement with the thickness *d* = 400 μm of the Li electrode, which is much larger than the skin depth *δ*_*mw*_ ≈ 1 μm for Li at 9.6 GHz^6,7^.

**Fig. 2:**
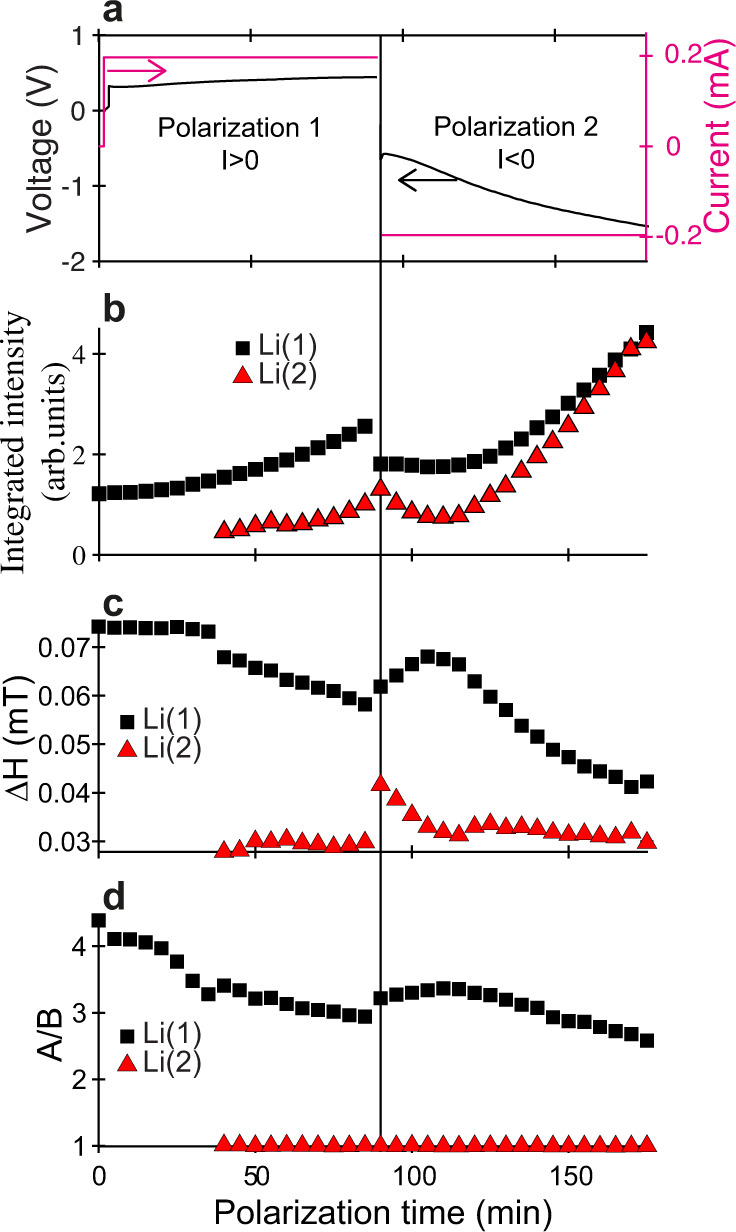
Evolution of the cw-EPR parameters versus polarization time for the two components, Li(1) (bulk Li) and Li(2) (micrometric particles). **a** Voltage (left) and current (right) of the electrochemical cell, **b** integrated intensity, **c** linewidth ΔH and **d** asymmetry ratio A/B.

The Li//Li cell was polarized to follow the mechanisms of Li stripping and plating with a current density of 1 mA/cm^2^ (Fig. 2a). During polarization 1, the top Li electrode is oxidized (Li stripping) and the bottom electrode is reduced (Li electroplating). In polarization 2 this process is reversed. No sign of cell failure was observed after 175 min of positive and negative polarization. Three spectra of the cell, corresponding to the pristine state (*t* = 0 min), the end of polarization 1 (*t* = 85 min) and the end of polarization 2 (*t* = 175 min), are shown in Fig. 1c. Between the two polarization steps, the cell was left in open circuit (OCV) to record in situ EPR images. In contrast, EPR spectra were recorded operando during the polarization steps, that is with current flowing through the cell. The spectra exhibit different lineshapes, indicative of a modification of the Li electrode morphologies upon polarization. The growth of a narrow and symmetric line in the low field flank of the bulk Li signal results in a splitting of the EPR spectrum (Fig. 1c). Each EPR spectrum was simulated by the sum of two contributions: (i) a dysonian line for bulk Li (Li(1)) with an asymmetry ratio 4 > A/B > 2, a fixed resonance field H_*res*_ = 349.15 mT and a varying linewidth ΔH; (ii) a narrow (ΔH ~ 0.03–0.4 mT) and symmetric (A/B ~ 1) lorentzian line (Li(2)) with resonance field H_*res*_ = 349.12 mT (corresponding to *g* = 2.0032), lower than for bulk lithium. This new EPR line is assigned to Li particles with size *d* < 1 μm, as discussed above. An example of simulation is given in Fig. 1d. The evolution of the EPR spectrum parameters for the two Li species (integrated intensity, linewidth ΔH and asymmetry A/B) during cell operation are given in fig. 2. The integrated intensities of the simulated lines continuously increase as the cell is polarized, to reach a factor 4 at the end of polarization 2 (*t* = 175 min), as shown in Fig. 2b. This increase in the integrated intensity is assigned to the growth of sub-micrometric Li particles (Li(2)) on both electrodes but also to the increased integrated intensity of the EPR line of bulk Li (Li(1)). The latter should not be misinterpreted as an increase in the total quantity of metallic Li during polarization. This increased integrated intensity of bulk Li signal (Li(1)) is the direct consequence of the progressive decrease of the asymmetry ratio A/B from 5 to less than 3 at the end of polarization 2 (Fig. 2d). This feature is the manifestation of the alteration of the two bulk electrode surfaces during cycling, which creates Li structures with size approaching the skin depth *δ*_*mw*_. As only electron spins in the volume of the skin are responsible for the EPR signal, the roughening of bulk lithium at constant total volume of metal induces an increase of the skin volume, and thus an increase of the integrated intensity. The decrease of ΔH (Fig. 2c) for bulk lithium during polarization is also the consequence of the progressive decrease of A/B for the bulk Li signal.

This evolution of the EPR signal during cell cycling indicates that the reaction Li^0^ → Li^+^ + e^−^ (1) at one electrode and the reverse reaction Li^+^ + e^−^ → Li^0^ (2) at the other electrode are not equivalent. In polarization 1, newly formed sub-micrometric Li particles are produced on the bottom electrode (Li_*b*_) with reaction (2), while the progressive roughening of the bulk Li electrode on top (Li_*t*_) occurs through reaction (1). It seems, however, that there is a beginning of reversible behavior in the first few minutes of polarization 2, when the current is reversed, with a decrease in the integrated intensity of both types of lithium, as well as an increase of the A/B ratio and the linewidth ΔH of bulk lithium (Fig. 2). This could indicate that the previously-formed Li microparticles on Li_*b*_ are partially consumed by reaction (1) and/or structures of lithium in electrode Li_*t*_ are fused together into larger structures through reaction (2). In the second part of polarization 2, however, the general evolution observed during polarization 1 continues, with an increase in the integrated intensities of both Li types, and a decrease of A/B and ΔH of bulk lithium (Fig. 2). This demonstrates the high sensitivity of in situ EPR spectroscopy to the nucleation of very small Li particles and the roughening of bulk Li electrodes, and places it as a tool of choice for studying the effect of prolonged cycling.

Interestingly, the resonance field H_*res*_ of EPR line for the sub-micrometric Li particles is shifted to low field by 0.03 mT compared to the bulk Li signal. Although the nature of this shift requires further studies, it is likely that it is produced by the dynamic nuclear polarization of metallic lithium particles via the Overhauser effect^12–14^: the partial saturation of the EPR line polarizes the nuclear spins, which creates a nuclear field H_*nuc*_ that may theoretically reach 0.03 mT, adding to the external field H_0_. As the spins resonate in the effective field H_*eff*_ = H_0_ + H_*nuc*_ > H_0_, the resonance condition, hf = g*μ*_*B*_(H_*res*_ + H_*nuc*_) requires that H_*res*_ decreases when H_*nuc*_ increases. This effect is maximum when the electron spins “experience” all the nuclear spins, as in the case of the sub-micrometric Li particles. A recent NMR investigation showed that dynamic nuclear polarization (DNP) of metallic lithium was a powerful technique for characterizing electrodeposited microstructures^15^. However, it appears more likely that this shift of 0.03 mT is the combination of nuclear polarization and a g-shift slightly higher than of bulk Li signal.

**Fig. 3:**
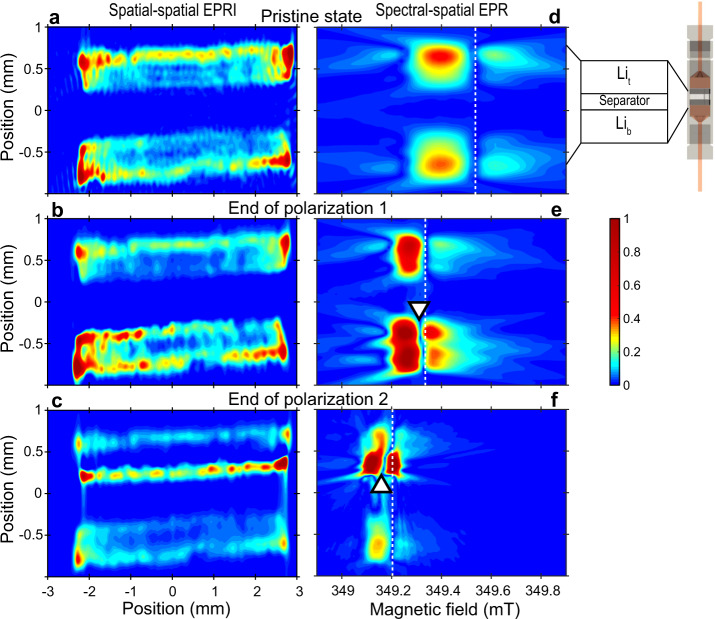
In situ X-band EPR images. **a**–**c** Spatial-spatial images and **d**–**f** spectral-spatial images of the cell in the pristine state, at the end of polarization 1 and at the end of polarization 2, respectively. The spectral-spatial images display the absolute value of the EPR line ∣*dχ”*/*dH*∣. The vertical dashed line indicates the resonance field of the bulk Li signal and the white triangle the resonance field of the sharper component (guides to the eye). The field of the vertical dashed line shifts between spectral-spatial EPR images due to a change of the microwave frequency. The color bar indicates the color code for the peak to peak amplitudes, identical for all plots. The illustration of the EPR cell has been created by Charles-Emmanuel Dutoit using an open-source 3D computer graphics software (Blender 3D).

In summary, the surface of the stripped Li electrode roughens during electrochemical polarization, with structures of micrometric size or slightly larger, and new Li particles of sub-micrometric size nucleate through electroplating on the opposite electrode. In order to visualize and locate these two types of metallic Li in the electrochemical cell, we performed in situ electron paramagnetic resonance imaging (EPRI) of the full cell (Fig. 3a–c). EPR images were recorded in the pristine state, at the end of polarization 1 and of polarization 2, in open-circuit phases to prevent compositional evolution during recording. First, pure two-dimensional imaging (spatial-spatial mode) was tested, with a gradient of 175 G/cm for encoding spatially the lithium signal. Thanks to the sharp EPR lines of metallic Li (<1 G) the EPR images can be obtained with a high spatial resolution of the order of micrometers in the pristine state^9,16^.

Spatial-spatial EPR images are shown in Fig. 3(left). They confirm that metallic Li is located only in the two electrodes initially (Fig. 3a). The separator inside the cell, with a thickness of 500 μm, appears in the center from the absence of EPR signal during all the electrochemical steps. Its apparent size is similar to the real size and is indicative of a relatively good alignment of the cell. In principle, one would expect to observe a similar peak to peak amplitude within the two electrodes in the pristine state. The observed contrast may have several physical origins, such as, for example, local variations of the microwave field caused by shielding or eddy current effects^9^. After the positive current flow (end of polarization 1, Fig. 3b), the peak to peak amplitude decreases in electrode Li_*t*_ (top). Additional spots corresponding to metallic Li deposition appear on electrode Li_*b*_ (bottom), and more particularly at the interface with the separator. The reverse phenomenon is observed after inversion of the current flow (end of polarization 2, Fig. 3c), with the metallic Li peak to peak amplitude increasing on the surface of Li_*t*_, near the separator, and an peak to peak amplitude decrease in Li_*b*_. This imaging mode, however, does not allow distinguishing clearly the two types of metallic Li.

In order to map the distribution of metallic lithium while preserving the spectroscopic (lineshape, resonance field) information, we turned to the correlation of spectroscopy and imaging (spectral-spatial EPR). Compared to classical spatial-spatial imaging, the spectral-spatial imaging gives access to parameters like the resonance field, the linewidth and the lineshape which are sensitive to the evolution of the electronic structure and the size of Li structures. The technique consists in scanning the whole cell with the field gradient (175 G/cm) applied along the **Y** direction (cell axis). The image (electron density profile) is reconstructed by stacking the EPR spectra obtained for each slice along the **Y** axis. In the resulting two-dimensional plots shown in Fig. 3 (right) the shape, resonance field and peak to peak amplitude of the EPR spectra can be determined for each slice along the **Y** cell axis. For clarity, the plot indicates the variation of the absolute value of the first derivative signal ∣*dχ”*/*dH*∣ with the external magnetic field. In this representation, the spectra consist in two lobes along the magnetic field separated by a narrow domain with zero peak to peak amplitude. The main features of the EPR spectra can be recognized: (i) the field splitting between the two lobes gives the peak-to-peak linewidth, ΔH of the EPR line, (ii) the relative peak to peak amplitude of the two lobes gives the asymmetry apparent ratio A/B, and (iii) the boundary between the two lobes is the value H_*res*_ of the external field H_0_ at which resonance occurs, as indicated by dashed lines and white triangles.

The spatial distribution of the two types of lithium was obtained more precisely with these spectral-spatial images. Initially, both electrodes show similar spectral characteristics, i.e., a broad signal with the low field lobe more intense than the high field one, revealing the presence of only bulk lithium foils (Fig. 3d). Several modifications are observed at the end of polarization 1 (Fig. 3e): (i) the EPR line is more intense in electrode Li_*b*_ than in electrode Li_*t*_, and reveals the submicrometric Li deposition on the bottom electrode, in agreement with spatial-spatial imaging. Interestingly (ii) the peak to peak amplitude of the top (depleted) electrode is higher than in the pristine state. This effect is the consequence of the roughening of the electrode surface upon stripping (increase of the volume probed within the skin depth), in good agreement with our interpretation of pure EPR spectroscopy (Fig. 2b). In addition, (iii) the linewidth of bulk Li decreases in both electrodes, in agreement with Fig. 2c. More importantly, in Fig. 3e (iv) H_*res*_ of the top part of the Li_*b*_ electrode is slightly shifted to lower field (white triangle) compared to the bottom part of the same electrode (vertical dashed line). This result indicates that the sub-micrometric particles appear at the interface with the separator and not in the bulk of the bottom electrode. This trend is magnified, though in the opposite direction, at the end of polarization 2 (current reversed) in Fig. 3f: (i) the bottom electrode is significantly depleted, while the signal of the top electrode is narrower, more intense and symmetrical at the interface with the separator; (ii) H_*res*_ of the bottom part of electrode Li_*t*_ (white triangle) is again shifted to lower field with respect to the top part (dashed line). These features indicate the nucleation of an increased number of sub-micrometric Li particles at the electrode-separator interface.

**Fig. 4:**
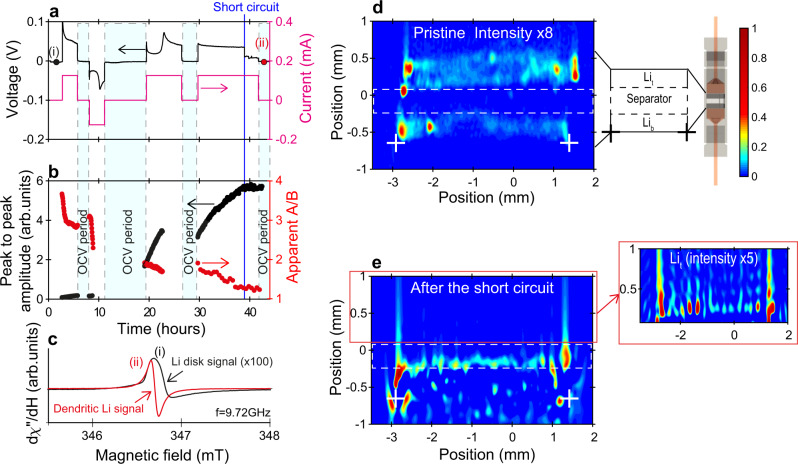
In situ detection (EPR spectrum) and localization (EPR images) of dendritic Li. **a** Voltage and current of the electrochemical cell versus time. The vertical blue line indicates the short circuit with a voltage drop to 0 V and current still flowing in the cell. **b** Operando peak to peak amplitude and apparent A/B ratio measured on the entire EPR signal (no simulation) as a function of time. 50% of the data are missing for the second and third cycling phase due to clipping effects from a non-optimal receiver gain. **c** EPR spectra of the cell recorded in the pristine state (bulk Li electrodes) and after the short circuit (dendritic Li). The resonance field is H_*res*_ = 346.82 mT for the bulk Li signal and H_*res*_ = 346.7 mT for the dendritic Li signal. **d**, **e** In situ spatial-spatial EPR images recorded in the pristine state (peak to peak amplitude x8) and after the short circuit, respectively. Spots crossing the separator after the short circuit suggest the location of dendritic Li. The inset shows a zoom in the region of the top electrode Li_*t*_, not fully consumed. The white crosses are a guide to the eye for the lower part of the bottom electrode. The color bar indicates the color code for the peak to peak amplitudes, identical for all plots. The illustration of the EPR cell has been created by Charles-Emmanuel Dutoit using an open-source 3D computer graphics software (Blender 3D).

Finally, let us discuss the well-known problem of dendritic lithium nucleation which ultimately leads to short-circuit. Figure 4 focuses on an in situ dendrite-induced short-circuit in another symmetric Li//Li cell. This phenomenon is visible in the galvanostatic profile in Fig. 4a. We detected a sudden drop to 0 V after almost 38 h of cycling with the current still flowing, which is indicative of a global short-circuit. The corresponding evolution of the peak to peak amplitude and apparent A/B of the entire spectrum are given in Fig. 4b. The peak to peak amplitude initially increases while apparent A/B decreases. This indicates a progressive increase in the number of electron spins submitted to the microwave field, a signature of sub-micrometric Li particles nucleation and of the roughening of the electrodes. After the short-circuit, however, the peak to peak amplitude no longer varies with time, in agreement with the failure of the cell. Figure 4c shows the EPR spectra recorded (i) in the pristine state (×100) and (ii) after the short-circuit. The broad and asymmetric lineshape of (i) corresponds to the bulk lithium electrodes. The second spectrum (ii) appears at lower field, symmetrical and more intense than (i) by a factor ~100. This intense and very sharp peak associated with the short-circuit corresponds to a large predominance of sub-micrometric Li particles, i.e., the modification of the morphology of the whole lithium disks surface. The bulk lithium signal is still present, but becomes negligible due to the already discussed higher sensitivity of EPR to the sub-micrometric Li particles. It is important to note that Li dendrites with sub-micrometric thickness may also contribute to this signal. This hypothesis is confirmed by the spatial-spatial EPR images recorded in the pristine state (Fig. 4d) and after the short-circuit (Fig. 4e). The presence of a dendrite is evidenced by a red spot on the right-hand side of Fig. 4e that crossed the separator and short-circuited the cell. Note that the top electrode was not consumed entirely; its peak to peak amplitude is lower as explained above, but we can detect it (inset in Fig. 4e). Additional red signals are dispersed under the bottom electrode (indicated by white crosses) and correspond to Li sub-micrometric particles deposited on the current collector after the short-circuit.

To conclude, we performed in situ EPR measurements on symmetric Li/LiPF_6_/Li cells in operating conditions. We establish operando EPR spectroscopy and in situ EPR spectral-spatial imaging as two highly complementary tools to follow the changing morphology of metallic lithium during stripping and to identify, localize and distinguish dendrites and sub-micrometric lithium particle during plating. Two main features were observed from the evolution of the EPR spectra and spectral-spatial EPR images of metallic Li in the electrochemical cell during polarization: (i) the nucleation of sub-micrometric Li particles at the interface between the separator and the electrodes; (ii) the progressive roughening of the electrodes into fragments of size slightly larger than 1 μm. We also provide an in situ EPR image of dendrites in the separator between the two electrodes, at the origin of a short-circuit of the electrochemical cell. To the best of our knowledge, spectral-spatial EPR imaging was never used to investigate such electrochemical devices and we hope that these results will pave the way to the combination of operando EPR spectroscopy and in situ spectral-spatial EPR imaging to diagnose new generations of lithium-ion batteries.

## Methods

### Electrochemical cell

The symmetric Li/LiPF_6_/Li electrochemical cells were assembled in the homemade EPR cell^6^ using an argon-filled glove box. The current collectors were copper disks on each side. The electrodes were metallic lithium disks with a typical thickness of around 400 μm and a diameter of 5 mm. One piece of porous microfiber mat (Whatman type GF/D) was used to separate the electrodes. The whole content of the EPR cell was soaked with electrolyte (1 mol/L LiPF_6_ in a mixture of ethylene and dimethyl carbonate in weight ratio 1:1, Merck). Galvanostatic control was performed at room temperature using a VSP galvanostat from Bio-Logic. The EPR cell was polarized (polarizations 1 and 2 in Fig. 1a) with a current density of 1 mA/cm^2^. The current was paused at the end of polarization 1 to record the images. EPR spectra were recorded continuously during polarization with a time resolution of 5 min (operando EPR spectroscopy). The same procedure was used for polarization 2, with a current of −1 mA/cm^2^. The second EPR cell was polarized with a current density of 0.63 mA/cm^2^ for 3 h and −0.63 mA/cm^2^ for 3 h. It was polarized positively again, with the same current as before, during 20 h to observe the nucleation of dendrites.

### in situ EPR

In situ continuous wave (cw) electron paramagnetic resonance experiments were carried out using a conventional X-band Bruker E500 spectrometer operating at around 9.6 GHz and room temperature. The microwave power supplied into the resonator was set to 4 mW in order to avoid saturation of the EPR signal. The 100 kHz modulation depth of the magnetic field was set to 0.1 mT or less to prevent distortion due to over-modulation. Conversion time and time constant were set to 40.96 ms and 20.48 ms, respectively. Cw-EPR spectra were fitted with one dysonian and one derivative of Lorentzian using Matlab. The resonance fields of bulk (dysonian) and sub-micrometric Li (Lorentzian) were fixed at 349.15 and 349.12 mT, respectively.

The spectral-spatial and spatial-spatial images were recorded with a field-of-view of 7 mm and gradient strength of 175 G/cm. The size of spatial-spatial images was 512 × 512 pixels resulting in a pixel size of 13.7 μm. The high resolution spatial-spatial EPR images were obtained after a deconvolution of the acquired projections under a magnetic field gradient from a signal recorded without gradient and a filtered back-projection. 140 projections were recorded for the spectral-spatial images with a spectral resolution of 1024 points and a pixel size of 13.7 μm in the spatial dimension. The high resolution spectral-spatial images were obtained from a filtered back-projection of the acquired projections.

## Data Availability

The dataset that support the findings of this study is available here: 10.17632/ggb454f3d5.1.

## References

[1] Liu, K., Liu, Y., Lin, D., Pei, A. & Cui, Y. Materials for lithium-ion battery safety. *Sci, Adv*. **4**, eaas9820 (2018). 10.1126/sciadv.aas9820 PMC6014713 29942858

[2] Waldmann T, Hogg BI, Wohlfahrt-Mehrens M (2018). Li plating as unwanted side reaction in commercial Li-ion cells—a review. J. Power Sources.

[3] Hatzell KB (2020). Challenges in lithium metal anodes for solid-state batteries. ACS Energy Lett..

[4] Liu Q (2016). Understanding undesirable anode lithium plating issues in lithium-ion batteries. RSC Adv..

[5] Foroozan T, Sharifi-Asl S, Shahbazian-Yassar R (2020). Mechanistic understanding of li dendrites growth by in- situ/operando imaging techniques. J. Power Sources.

[6] Sathiya, M. et al. Electron paramagnetic resonance imaging for real-time monitoring of li-ion batteries. *Nat. Commun.* 6, 6276 (2015). 10.1038/ncomms7276 PMC4347297 25662295

[7] Wandt J (2015). Operando electron paramagnetic resonance spectroscopy—formation of mossy lithium on lithium anodes during charge–discharge cycling. Energy Environ. Sci..

[8] Wandt J, Jakes P, Granwehr J, Eichel R-A, Gasteiger HA (2018). Quantitative and time-resolved detection of lithium plating on graphite anodes in lithium ion batteries. Mater. Today.

[9] Niemöller A, Jakes P, Eichel RA, Granwehr J (2018). EPR Imaging of metallic lithium and its application to dendrite localisation in battery separators. Sci. Rep..

[10] Dyson FJ (1955). Electron spin resonance absorption in metals. ii. theory of electron diffusion and the skin effect. Phys. Rev..

[11] Feher G, Kip AF (1955). Electron spin resonance absorption in metals. i. experimental. Phys. Rev..

[12] Overhauser AW (1953). Polarization of nuclei in metals. Phys. Rev..

[13] Vigreux C, Binet L, Gourier D (1998). Bistable conduction electron spin resonance in metallic lithium particles. J. Phys. Chem. B.

[14] Vigreux C, Loiseau P, Binet L, Gourier D (2000). Anomalous metallic lithium phases: identification by ESR, ENDOR, and the bistable Overhauser effect. Phys. Rev. B.

[15] Hope MA (2020). Selective NMR observation of the SEI-metal interface by dynamic nuclear polarisation from lithium metal. Nat. commun..

[16] Maresch GG, Mehring M, Emid S (1986). High resolution ESR imaging. Physica B+C.

